# An Emergency-Adaptive Routing Scheme for Wireless Sensor Networks for Building Fire Hazard Monitoring

**DOI:** 10.3390/s110302899

**Published:** 2011-03-04

**Authors:** Yuanyuan Zeng, Cormac J. Sreenan, Lanny Sitanayah, Naixue Xiong, Jong Hyuk Park, Guilin Zheng

**Affiliations:** 1 School of Electronic Information, Wuhan University, China; 2 Mobile & Internet Systems Laboratory, School of Computer Science & Information Technology, University College Cork, Cork, Ireland; E-Mails: cjs@cs.ucc.ie (C.J.S.); ls3@cs.ucc.ie (L.S.); 3 Department of Computer Science, Georgia State University, USA; E-Mail: nxiong@cs.gsu.edu; 4 Department of Computer Science and Engineering, Seoul National University of Technology, Korea; 5 School of Power and Mechanical Engineering, Wuhan University, China; E-Mail: glzheng@whu.edu.cn

**Keywords:** wireless sensor networks, building fires, real-time, power adaptation

## Abstract

Fire hazard monitoring and evacuation for building environments is a novel application area for the deployment of wireless sensor networks. In this context, adaptive routing is essential in order to ensure safe and timely data delivery in building evacuation and fire fighting resource applications. Existing routing mechanisms for wireless sensor networks are not well suited for building fires, especially as they do not consider critical and dynamic network scenarios. In this paper, an emergency-adaptive, real-time and robust routing protocol is presented for emergency situations such as building fire hazard applications. The protocol adapts to handle dynamic emergency scenarios and works well with the routing hole problem. Theoretical analysis and simulation results indicate that our protocol provides a real-time routing mechanism that is well suited for dynamic emergency scenarios in building fires when compared with other related work.

## 1. Introduction

In the near future, it can be expected that buildings will be equipped with a range of wireless sensors functioning as part of an overall building management system. Included in this set of sensors will be devices to monitor fire and smoke, allowing detection, localization and tracking of fires. It is expected such information could be used for a variety of purposes, including guiding building occupants to the nearest safe exit, and helping fire fighting personnel to decide on how to best tackle the disaster. Fire/smoke sensors are expected to be programmed to report periodically and also when they detect a sensor input that exceeds a threshold. In the latter case, there is a need for an emergency-adaptive, real-time and robust message delivery toward the sink. For example, a fire-fighter relies on timely temperature updates to remain aware of current fire conditions. In addition, as the fire spreads throughout the building, it becomes likely that the sensing devices may become disconnected from the network or indeed be destroyed, so the network routes have to be changed or re-discovered to adapt to these emergency conditions in order for the network to continue operating. Most existing routing protocols consider the energy efficiency and lifetime of the networks as the foremost design factor. The routing mechanisms used in general wireless sensor networks and even routing for forest fire applications are not well suited for in-building disaster situations, where timeliness and reliability are much more critical. For forest fires the focus is on tracking of fires, rather than evacuation or guidance of fire personnel. This combination of real-time requirements coupled with dynamic network topology in a critical application scenario provides the motivation for our research. In this paper, we propose an emergency-adaptive routing mechanism (EAR) designed especially for building fire emergencies using wireless sensor networks (WSN), which provides timely and robust data reporting to a sink. We do not need to know the exact localization of each sensor and also no time synchronization is needed. To the best of our knowledge, this is the first time a real-time and robust routing mechanism adaptive to building fire emergency using WSNs has been proposed. Also, this protocol could be easily used in other similar emergency applications.

Section 2 presents the related work. In Section 3 we outline the routing problem. We present an emergency-adaptive routing mechanism in Section 4. In Section 5, we present a preliminary analysis. In Section 6, we give ns2 simulation results. Finally, Section 7 concludes this paper.

## 2. Background and Related Work

Most routing protocols for WSNs focus on energy efficiency and link node lifetime related explicitly to its energy resources, *i.e.,* a node is assumed to fail when the battery is depleted. Some WSN applications require real-time communication, typically for timely surveillance or tracking. Real-time routing protocols in WSNs are not new. For example, SPEED [1], MM-SPEED [2], RPAR [3] and RTLD [4] were all designed for real-time applications with explicit delay requirements. He *et al.* [1] proposed an outstanding real-time communication protocol binding the end-to-end communication delay by enforcing a uniform delivery velocity. Felemban *et al.* proposed [2] a novel packet delivery mechanism called MMSPEED for probabilistic QoS guarantee. Chipara *et al.* proposed [3] a real-time power aware routing protocol by dynamically adapting transmission power and routing decisions. But these routing protocols are not well suited for routing in emergency applications such as building fires, where critical and dynamic network scenarios are key factors. Amed *et al.* proposed [4] a novel real-time routing protocol with load distribution that provides efficient power consumption and high packet delivery ratio in WSN.

There are many robust routing protocols proposed for WSNs. Zhang *et al.* [5] proposed a framework of constrained flooding protocols. The framework incorporates a reinforcement learning kernel, a differential delay mechanism, and a constrained and probabilistic retransmission policy. The protocol takes the advantages of robustness from flooding. Deng *et al.* [6] presented a light-weight, dependable routing mechanism for communication between sensor nodes and a base station in a wireless sensor networks. The mechanism tolerates failures of random individual nodes in the network or a small part of the network. Boukerche *et al.* [7] presented a fault tolerant and low latency algorithm, which refer to as periodic, event-driven and query-based protocol that meets sensor networks requirements for critical conditions surveillance applications. The algorithm uses a publish/subscribe paradigm to disseminate requests across the network and an ACK-based scheme to provide fault tolerance. In building fires, network topology changes rapidly because of hazard and node failure, so general robust protocols are not suitable for such scenarios. Here, we want to design protocols that can be adaptive to the occurrence of fire, expanding, shrinking or diminishing, *etc.* So, “robustness” in this paper means “adaptive to fire situations”.

In this regard, the work by Wenning *et al.* [8] is interesting, as they propose a proactive routing method that is aware of the node’s destruction threat and adapts the routes accordingly, before node failure results in broken routes, delay and power consuming route re-discovery. They pay attention to the aspect of node failures caused by the sensed phenomena themselves.

However, in their work, they focus on disasters such as forest fire that are very different from design issues in building situations. Fire emergencies using wireless sensor networks within buildings are more challenging because of the complex physical environment and critical factors of fire hazards. In [9], we proposed a fire emergency detection and response framework for building environments using wireless sensor networks. We presented an overview of recent research activity including fire detection and evacuation, in addition to providing a testbed especially designed for building fire applications. Other researchers have worked on emergency guidance and navigation algorithms with WSNs for buildings. Tseng *et al.* [10] proposed a distributed 2D navigation algorithm to direct evacuees to an exit, while helping them avoid hazardous areas. Their design allows multiple exits and multiple emergency events in the sensing field. Sensors are used to establish escape paths leading to exits that are as safe as possible. When surrounded by hazards, sensors will try to guide people as far away from emergency locations as possible. Based on this, Pan *et al.* [11] proposed a novel 3D emergency service that aims to guide people to safe places when emergencies happen. In their work, when emergency events are detected, the network can adaptively modify its topology to ensure transportation reliability; quickly identify hazardous regions that should be avoided and find safe navigation paths that lead people to exits. Barnes *et al.* [12] presented a novel approach for safely evacuating persons from buildings under hazardous conditions. A distributed algorithm is designed to direct evacuees to an exit through arbitrarily complex building layouts in emergency situations. They find the safest paths for evacuees taking into account predictions of the relative movements of hazards, *i.e.,* fires and evacuees. Tabirca *et al.* [13] solve a similar problem, but under conditions where hazards can change dynamically over time.

When fire expands in an inner building, there may cause a lot of segmentation in the network. In this case, a lot of routing holes occur that lead to data routing failure. The “Routing Hole Problem” is a very important and well-studied problem, where messages get trapped in a “local minimum”. Some existing “face routing” algorithms have been developed to bypass routing holes using geo-routing algorithms. GPSR [14] recovers holes by using the “right-hand rule” to route data packets along the boundary of the hole, combining greedy forwarding and perimeter routing on a planar graph. The authors of [15] proposed the first practical planarization algorithm with a reasonable message overhead, lazy cross-link removal (LCR). Fang *et al.* [16] presented an interesting approach, the BOUNDHOLE algorithm, which discovers the local minimum nodes and then “bounds” the contour of the routing holes. In the building fire situation, holes feature prominently and can be expected to grow in size rapidly as a fire spreads, thus demanding solutions that are robust and low complexity for quick reactions.

## 3. Definitions

Given a homogeneous WSN deployed in a building for fire hazard applications with *N* sensors and *M* sinks, each sensor can adjust its maximal transmission ranges to one of the *k* levels: *r_0_*, *r_1_*… *r_k−1_* = *r_max_* by using different transmission power levels from *p_0_*, *p_1_*, till *p_k−1_* = *p_max_*. Initially, all sensors work in *p_0_*. From the application aspect, real-time and robustness are two main challenges. *T_max_* is the maximum acceptable delay in reporting such a fire event to a sink node. It is required that each sensor *i* will report data packets to a sink node, such that:

1. A communication path from sensor to the sink can be found if such a path exists.
2. The end-to-end delay of the path is no more than *T_max_*.
3. The choice of route is adaptively changed in response to failed nodes (assumed to be caused by fire damage).
4. A suitable minimized power level (*min* {*p_0_*, *p_1_*… *p_k−1_*}) is selected to ensure transmission to satisfy (1), (2), (3) without unnecessary power dissipation.

Each node in the network exists in one of four states (listed in the order of health degree from best to worst):

“*safe*”: initial state while no fire occurs.
“*lowsafe*”: one-hop away from an “*infire*” node.
“*Infire*”: when detects fire.
“*unsafe*”: detects that it cannot work correctly any longer due to a definite fire

There is a STATE message recording current change of node state to notify its neighborhood nodes in a fire.

STATE (INFIRE) message: If a sensor detects fire, it enters “*infire*” by broadcasting a message out to denote a new local fire source.

STATE (LOWSAFE) message: The nodes in “*safe*” state that receive a STATE (INFIRE) message will become “*lowsafe*”, and then broadcast a STATE (LOWSAFE) message to notify its neighbors. The nodes that hear the STATE (LOWSAFE) message will get to know the new state of its neighbors about fire and do nothing.

STATE (UNSAFE) message: An “*infire*” node works until it cannot work correctly. Before it cannot work any longer, it enters into “*unsafe*” state and broadcast a message. Any nodes that detect its residual energy is too low to work will enter into “*unsafe*”. And then broadcast a STATE (UNSAFE) message.

**Figure 1.**
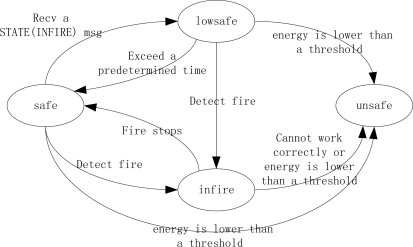
State transition diagram for each node.

Thus each sensor may change its state autonomously in response to the fire and messages it receives, as shown in Figure 1.

## 4. Protocol Description

### 4.1. Initialized Routing Structure

#### Initialized Sink Beacon:

The purpose of routing initialization is to form an initialized neighborhood and routing construction after the sensors are deployed and connected as a WSN in the building. We assume that sinks are deployed in a relatively safe place such that they are less likely to be destroyed, for example due to walls collapsing. Once the network is deployed, each sink generates a HEIGHT message using power level *p_0_*. This serves to advertise to neighbor nodes and includes a “*height*” parameter that represents the hop count toward the sink, and is initialized to 0. The height value is incremented by each forwarding hop. Each node records the height information in its local neighborhood table when it receives the first HEIGHT message. The message contains a sequence number so that a node can determine if it has seen the message already, in which case it ignores it. If it is the first time that it receives a HEIGHT message, the node forwards the HEIGHT message out. As explained below this process serves to ensure that each node will know a minimal delay route path from itself toward one of the sinks.

#### End-to-End HEIGHT Delay Estimate:

In this HEIGHT message broadcasting process, the end-to-end delay from a node to the sink could be approximated by the cumulative delay on each hop. We use “delay estimate” in our EAR routing mechanism to make the forwarding choice. We denote *delay* (*sink*, *i*) as the delay experienced from the sink to each node, and then we could use *delay* (*sink*, *i*) as a bound to guide a real-time delivery from the node to the sink. The delay in transmitting a packet is estimated by:

(1) $$\mathrm{delay}\left(\text{sink},i\right)=\sum_{n=1}^{h}\mathrm{Avg\_delay}=\sum_{n=1}^{h}\left(T_{c}+T_{t}+T_{q}\right)*R$$

In formula (1), *n* is the hop count from the sink to node *i*, *T_c_* is the time it takes for each hop to obtain the wireless channel with carrier sense delay and backoff delay. *T_t_* is the time to transmit the packet that determined by channel bandwidth, packet length and the adopted coding scheme. *T_q_* is the queuing delay, which depends on the traffic load, and *R* is the retransmission count. Among them, we omit the propagation delay, as in a WSN this is negligible due to the use of short-range radios. In the delay calculation, the delay of MAC layer with MAC protocol used is counted in.

The average end-to-end delay from each node to the sink can be computed as the cumulative hop-by-hop delay, and the delay experienced in the current hop is calculated and updated locally, and then recorded in the HEIGHT message. Then *delay* (*sink*, *i*) is recorded into the neighborhood table of each node. We use a periodic HEIGHT message update to calculate an average end-to-end delay (from multiple end-to-end delay estimates) as reference. Since packets in WSNs always tend to be relatively small, we consider it reasonable to ignore any impact of delay differences related to packet size. Furthermore, delay estimate utilizes Jacobson’s algorithm [17] to make adjustment by considering both the weighted average and variation of the estimated variable and as a result provides a good estimate of the delay. It can work well when link quality and network load varies. The calculation of average end-to-end delay and variation avoids a large number of deadline misses due to high variability in communication delays.

Since the traffic from the node to the sink is usually heavier than the traffic from the sink to the node under the same radio situation according to sensor applications, we can say that queuing delay *T_q_*(*sink*, *i*) ≤ *T_q_*(*i*, *sink*). This is bounded by the maximum queuing delay, *i.e., T_q_*(*i*, *sink*) ≤ *T_qmax_*. When assuming the same radio and link quality for downstream and upstream links on the counterpart route path, we can get that: *delay* (*i*, *sink*) ≤ *delay_qmax_*(*sink*, *i*). *delay_qmax_*(*sink*, *i*) is the delay experienced from sink to *i* with the maximal delay on queuing. Then our delay estimate and realistic delay on the route path *T* satisfy: *delay* (*sink*, *i*) ≤ *T* ≤ *delay_qmax_*(*sink*, *i*). We can use *delay* (*sink*, *i*) as a “bound” to guide the real-time routing forwarding selection. If the delay and *slack* time (defined as time left for routing) meets the estimated delay time for data delivery, the packet has a high probability to arrive before deadline and thus ensures real-time communications.

#### Periodic Sink Update:

With the HEIGHT message broadcast process, an initial neighborhood is formed by each sensor for which it records neighbor ID, height, state, estimated delay, residual energy of all neighbors, as well as the transmission power that the node uses to communicate with its neighbor on the path to the sink. Each sensor records its own ID, state, and residual energy. In addition, each node maintains sink ID with its minimal-delay sink. In a fire scenario, the sink may become disabled and the network’s topology will be changed by the fire. To ensure robust connectivity, each sink will periodically send out a HEIGHT message to refresh the network. The refresh rate is a protocol design parameter that trades off overhead for increased robustness to lost HEIGHT messages and path changes. In a fire situation one would expect to decrease the period, although the impact on network traffic load must also be examined.

### 4.2. Routing Mechanism Details

#### Forwarding Choice:

For a given application-specific *T_max_*, we use *slack* to remember the time left on the path from the current node to the sink. Each node in the neighborhood table is associated with a *forward_flag* and a *timeout*. The flag is used to identify the next hop as a best forwarding choice, *i.e.,* when a node is chosen as the best forwarding choice, the *forward_flag* is set to 1. The *timeout* value is the valid time for the current forwarding node and used to prevent stale neighborhood information (introduced in Section 4.3.) If “*timeout*” of a forwarding choice is due, its forwarding flag is set to 0 to evict the stale relay node.

To select the best forwarding choice from local neighborhood table, we use the following criteria:

Firstly, we filter the forwarding choices by “*height*” to choose the nodes with lower height.
Secondly, choose the node with enough *slack* time according to delay estimate on the path.
Thirdly, we filter the remaining forwarding choices by node state in the priority from “*safe*” to “*infire*”.

If there is more than one node satisfied, we select the best forwarding choice with higher residual energy. If there is still a tie, we choose the lower ID.

If we cannot find a best forwarding choice with the current transmission power, we say that a “hole” has occurred (*i.e.,* stuck in local minimum).

#### Hole Problem Handler by Adapting Power level:

If a sensor node cannot find a next hop that satisfies the real-time constraint with current power level, it means that the node is stuck in a local minimum. The solution is to increase the transmission power gradually by levels to find another neighbor or invoke a new neighbor discovery. Otherwise, a notify message is sent back to its upstream node (*i.e.,* parent) to stop sending data packets to the current node; and then a routing re-discovery is invoked by the upstream node.

**Figure 2.**
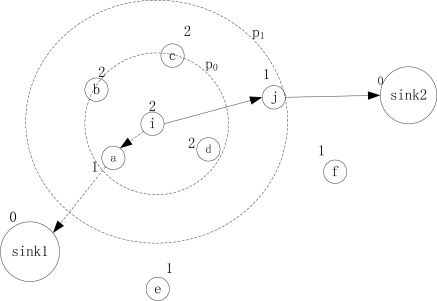
New neighbor discovery to solve routing “hole”.

If we could find another node existing in the neighborhood table by adapting the transmission power, then we increase the power level and name this neighbor as a forwarding choice. Otherwise, a new neighbor discovery is invoked by increasing the transmission power gradually by levels. We increase power level gradually but not to the maximal power level directly by considering of the big interference incurred by larger power. We know that there are only two to three power levels provided on existing MICA motes and most of the motes currently used. So, it converges very quickly to the optimal power level. Figure 2 shows an example of a new neighbor discovery, where *sink1* and *sink2* are two sinks, and the other nodes are sensors. Node *i* reports and routes data to the sink. The number on each node represents the “*height*” of each node toward the sink. As the route path {*i*, *a*, *sink1*} with *p_0_* is invalid because *slack* cannot satisfy the estimated end-to-end delay, node *i* is in the “hole”. If there are no existing eligible neighbors, then *i* will increase its power to *p_1_* to reach node *j* and delivers the packets to another sink *sink2* by route path {*i*, *j*, *sink2*} when “*slack*” on this route is no less than delay estimate.

Each sensor has *k* levels of power setting: {*p_0_*, *p_1_*, *p_2_*…*p_k−1_*} and could be in *k* levels of maximal transmission range as: {*r_0_*, *r1*…*r_k−1_*}. We defined a function to find appropriate transmission power by increasing the power as follows:

(2) $$p=p_{\mathrm{cur}+\iota +1},\iota =1,2,3\ldots .k-1$$

where, *cur* is the current number of transmission range level among *k* levels, *ι* is the count of unsuccessful attempts. A sensor will increase its transmission power gradually in levels if it cannot find an eligible new neighbor.

A node increases its power according to formula (2) until one of the following conditions is satisfied:

1. It finds a node as a forwarding choice in “*safe*” state according to the height and delay estimate.
2. If *p* = *p_max_*; in this case, it finds the new neighbor as a forwarding choice by the height and delay estimate in a priority from “*safe*”, “*lowsafe*” to “*infire*”; otherwise, no eligible new neighbor can be found.

In the new neighbor discovery, sensor *i* will broadcast out a Routing Request (RTR) message. In this process, sensor *i* piggybacks *height*, *slack* and the newly adapted power *p_i_* in RTR message. For a node *j* that hears the message, if the estimated end-to-end delay is no more than *slack* and its height is lower than *height*(*i*, *sink*), as well as its state is “*safe*”, then *j* is selected as a new neighbor. If sensor *j* hears the RTR with *p_max_*, and if its height is lower than *height* (*i*, *sink*), then *j* is selected a new neighbor when *j* is not in “*unsafe*” state. The new neighbor will reply to node *i* with the same power that node *i* is using, after a random backoff to avoid collisions. The forwarding choices send reply message with *p_i_* only as necessary for reaching node *i*, otherwise reverting to their previous power level. Upon receiving the reply, node *i* inserts the new neighbors into its neighbor table. During RTR and reply message exchange, we could calculate the delay between *i* and its new neighbor *j* as follows:

(3) Ave_delay(i,j)=Round_trip_time/2

For meeting real-time requirements, the forwarding choices should satisfy that: “*slack*” is no less than the average delay between *i* and *j* plus the delay estimate at node *j*, *i.e.,*

(4) slack(i)⩾Ave_delay(i,j)+delay(sink,j)

If there is more than one new neighbor found, a best forwarding node is selected by the priority of state from “*safe*”, “*lowsafe*” to “*infire*”. If there is still a tie, the best relay is selected by the node with higher residual energy and lower ID number.

For a node that works in a larger transmission range could still be adapted to decrease the transmission power to improve energy efficiency and network capacity, while delay deadline is loose. So we define when a node detects a good connectivity with safe neighborhood that is larger than a predefined threshold, *i.e.,* |*Neighbor_safe_*| > *N_threshold*, power decrease process is invoked.

We defined a function to find appropriate transmission range by decreasing transmission power as follows:

(5) $$p=p_{\mathrm{cur}-\iota \prime ,}\iota =1,2,3\ldots .k-1$$

In formula (5), *cur* is the current number of transmission power level among *k* levels. *ι′* is the count of decrement.

A node is eligible for power decrease until:

1. The minimum power has been reached.
2. There are two consecutive power levels such that at the lower level the required delay is not met but at the higher power level the required delay is met.
3. There are two consecutive power levels such that at the lower level the required safe neighborhood connectivity *N_threshold* is not met but at the higher power level it is.

#### Neighborhood Table Management:

The neighborhood table records information including transmission power for reaching the neighbor nodes, and is updated by periodic HEIGHT messages from sinks. For power adaptation and new neighbor discovery, the neighborhood table will be updated with the new neighbors and new transmission power. The node also updates its neighborhood with the neighbors and new states as they change. If it receives a STATE (UNSAFE) message, the unsafe neighbor is removed from the table.

### 4.3. Routing Reconfiguration

In building fire emergencies, robust routing is crucial due to the impact of quickly moving fire on node liveness. In this section, we explain how we reconfigure to deal with failures. We assume that: (1) the minimal time interval between “*infire*” and “*unsafe*” state of a node is chosen as a parameter known beforehand and denoted as *t_unsafe_*. (2) We use necessary transmission range for connectivity between nodes (according to selected power level) to approximate the minimal fire spreading time between two nodes. In practice, there are well-known guidelines for estimating the rate of fire spread [18–19], taking into account of building materials, building geometry, *etc.* It’s also the case that obstacles, such as walls, that mitigate radio propagation also have the effect of slowing fire spread.

When a forwarding choice is used for relaying, we add “*timeout*” and avoid using stale and unsafe paths, *i.e.,* every node on the path from source *s* to destination *d* has “*timeout*” to record the valid time of each link on this route. The *timeout* is updated when node state changes occur among the neighborhood. The forwarding choice that exceeds the *timeout* value is considered invalid and then evicted.

We assign an initialized large constant value to represent the estimated valid time for the node in “*safe*” state.

When a neighbor node *j* is caught in fire, a STATE (IN-FIRE) message is broadcast. If a “*safe*” node *i* receives a STATE (IN-FIRE) message from its neighbor, node *i* will enters into the “*lowsafe*” state. The timeout of node *i* is updated, *i.e.,* the valid time of node *i* is updated, as the minimal time that this node may be caught in fire until it is out of function:

(6) $$\mathrm{timeout}\;(i)=\text{min}(\mathrm{spread\_time}(i,j)+t_{\mathrm{unsafe}}$$

Then the *timeout* value of both downstream and upstream links that are adjacent to node *i* are also updated accordingly. If node *i* becomes “*infire*”, the *timeout* of adjacent links are updated as *t_unsafe_*, *i.e., timeout* (*i*) = *t_unsafe_*.

Otherwise, if node *i* becomes “*unsafe*” by local sensed data and threshold, then *timeout* (*i*) is updated as 0 and the *timeout* of the adjacent links are also updated to 0.

The link *timeout* value is updated as the state of the node adjacent to the link changes. When a node state is changed for fire, the “*timeout*” on upstream and downstream links that are adjacent to this node will both be updated. For path link (*i*, *j*) on each route path, the *timeout* value for this link is calculated as:

(7) timeout (link(i,j))=min(timeout(i), timeout(j))

In formula (7), *timeout* (*i*) and *timeout* (*j*) represents the valid time for node *i*, *j* of the route in fire, respectively.

In a building fire, node failures because of fire damage will trigger routing tree reconfiguration. In case of a path link *timeout* value that is lower than a threshold (*i.e.,* the route path will be invalid very soon), a route reconfiguration is invoked to find another available route path before the current one becomes invalid. The reconfiguration is only invoked by an upstream node *i* of the path link (*i*, *j*) whose valid time is no less than the timeout of the link, *i.e., timeout* (*i*) ≥ *timeout* (*link* (*i*, *j*)). The routing reconfiguration of the node is invoked as a routing re-discovery by broadcasting a RTR message to set up a new route path search. The search of the forwarding choice is invoked in its neighborhood table to find if one of the existing neighbors is eligible to act as a relay or not by adapting the power to the setting recorded in local neighborhood. Otherwise, we will start a neighbor re-discovery process by increasing its power level gradually.

The re-discovery process stops when it finds another new forwarding choice with a valid route path cached toward one of the sinks (that could be a different sink from current one).

**Figure 3.**
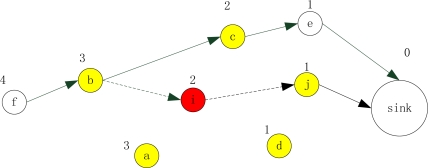
Timeout update in fire and route reconfiguration.

Figure 3 shows an example for timeout update in fire. For sensor *f*, it reports to sink by route path: {*f*, *b*, *i*, *j*, *sink*}. After working for a while, sensor *i* (colored red) senses the fire occurrence. Then sensor *i* broadcasts a STATE (IN-FIRE) message to notify its communication neighbors (colored yellow): *a*, *b*, *d*, *j*, and *c*. When these nodes receive the message, they will enter into the “*lowsafe*” state. For the state change of sensor *i*, then *timeout* (*i*) is updated as *t_unsafe_*. Accordingly, sensor *i* will update the timeout of its upstream and downstream link, *i.e., link* (*b*, *i*) and *link* (*i*, *j*). As our designed condition for reconfiguration, when *timeout* (*link* (*b*, *i*)) and *timeout* (*link* (*i*, *j*)) is lower than a predetermined threshold, the routing reconfiguration is invoked by the upstream node whose timeout is no less than the link timeout. Then sensor *b* will broadcast a RTR message to find a new relay to the sink, *i.e.,* route path {*f*, *b*, *c*, *e*, *sink*}. When it comes to path link (*i*, *j*), sensor *i* is the upstream node of the link with the lower valid time. It will still work on this path (to forward data from sensor *i* to the sink) until sensor *i* becomes “*unsafe*”.

It is assumed that data packet acknowledgements are sent at the link layer (not end-to-end). When a node does not receive an acknowledgement after a given time, we say the downstream link becomes invalid and then reconfigure routing.

## 5. Analysis

***Lemma1.*** The EAR routing of the sensor network graph is loop-free.

Proof: Suppose that there exists a loop “A→B→C→D→E→⋯→A” in the network graph by EAR routing. Each node selects its next node which has less height towards the sink. When a node is stuck in local minimum, *i.e.,* in a routing hole, the node could increase its transmission range to find another node that has less height towards the sink if exists. According to this, we could get: *height*(A) <⋯< *height*(E) < *height*(D) < *height*(C) < *height*(B) < *height*(A). This is a contradiction, so we conclude that the EAR routing of the network graph is loop-free.

***Theorem 1.*** If there exists a route within delay bound from a node to one of the sinks, EAR can find this route.

Proof: From Lemma 1, we know that there is no loop in the routing graph. Since the number and height of sensor nodes is limited, so the routes will lead to the sink eventually as long as the real-time route exists.

***Theorem 2***. For a given delay bound *T_max_*, the routing path found by EAR is within the delay requirement.

Proof: We denote *delay* (*sink*, *i*) as the delay estimate that is the minimal delay from the sink to the node, while *delay* (*i*, *sink*) as the delay from the node to the sink on the counterpart route path. We denote *T* (*i*, *sink*) as the realistic delay experienced from a node to the sink. For queuing delay in wireless sensor networks, data packets are always reported from the node to the sink, while less traffic (usually control command) is delivered from the sink to the node. So *T_q_*(*sink*, *i*) ≤ *T_q_*(*i*, *sink*). When assuming the same link quality of upstream and downstream links, there exists: *delay* (*sink*, *i*) ≤ *delay* (*i*, *sink*) ≤ *T* (*i*, *sink*). In EAR, we use *delay* (*sink*, *i*) as estimate of delay time form the node to the sink in routing discovery to find a route that meets the lower delay threshold, *i.e.,* using *delay* (*sink*, *i*) to estimate *T* (*i*, *sink*). In this way, we could improve the real-time delivery ratio from the node to the sink. Since we measure the average delay with HEIGHT using power *p_0_*, we get the maximal delay estimate time *delay* (*sink*, *i*) on the minimum delay route path from the sink to the node within different power levels. In EAR, we find a relay node *i* that delay *T* from *i* to the sink with this route path should satisfy that it is no larger than the delay estimation on the route path, *i.e., T* (*i*, *sink*) ≤ *delay* (*sink, i*). Otherwise, we increase the power level to find another forwarding choice *j*, and such a node *j* (with increasing power) exists by satisfying: *delay* (*sink, j*) + *Ave_delay* (*i*, *j*) ≤ *T_slack_*; where *T_slack_* = *T_max_* – *T*(*s*, *i*). The end-to-end delay time *T* satisfies: *T*(*s*, *sink*) = *T*(*s*, *i*) + *T*(*i*, *sink*) ≤ *T*(*s*, *i*) + *Ave_delay*(*i*, *j*) + *delay*(*sink, j*) ≤ *T*(*s*, *i*) + *T_slack_* ≤ *T_max_*. So, we find a route from node *s* to the sink that satisfies *T*(*s*, *sink*) ≤ *T_max_*.

From the above situations, if a real-time route exists, EAR can find a route path satisfying that the end-to-end delay is within the delay requirement *T_max_*.

## 6. Simulations

**Figure 4.**
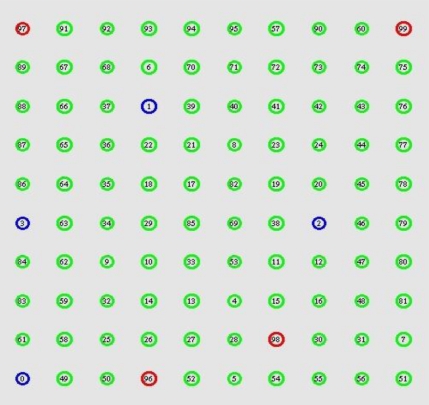
Simulation grid.

We verify our routing by simulations using the ns2 network simulator [20]. To create a realistic simulation environment, we simulated EAR based on the characteristics and parameters of the MICAz motes, as shown in Table 1. All nodes could be used to work with three power levels and they will work in the minimal power level as the default transmission power. Many-to-one traffic pattern is used, which is common in WSN applications. This traffic is typical between multiple source nodes and one of the sinks. There are 100 nodes distributed in a 100 m × 100 m region as shown in Figure 4. We randomly select four nodes as source nodes, and place 1–4 sinks in the simulation areas as node 99, 98, 97 and 96 respectively. Each source generates constant bit rate (CBR) traffic periodically. The real-time packet miss ratio and packet dismiss ratio by delay estimate as well as energy consumption are assigned as the main metrics for evaluating the performance of EAR. The real-time packet miss ratio (we use “miss ratio” in the following paragraph) is the ratio of all packets missed because of the delay bound to the total packets sent out.

The packet dismiss ratio by delay estimate (we use “dismiss ratio” in the following paragraph) is defined as the ratio of packets discarded by delay estimate and the total sending packets. The energy consumption is the average energy consumed for each sensor during the simulation. Within the simulated area, a fire breaks out 30 seconds after the simulation is started which means the first 30 seconds of the simulation. The node in the network is static. At 30 seconds after the simulation begins, a fire occurs randomly in the network area and then spreads to its neighbors continuously every 10 seconds. When the fire reaches a sensor node, it will lead to a terminal node failure after 10 seconds.

We compare our protocol with minimal hop count routing and RPAR protocol to make performance evaluation. The two comparing routing mechanisms are operated with the initial power as the default transmission power in EAR. RPAR is a real-time power-aware routing mechanism that achieves this by dynamically adapting transmission power and routing decisions based on packet velocity calculated by geographical distance and time left.

### 6.1. EAR Performance When Sink Number Increases

**Figure 5.**
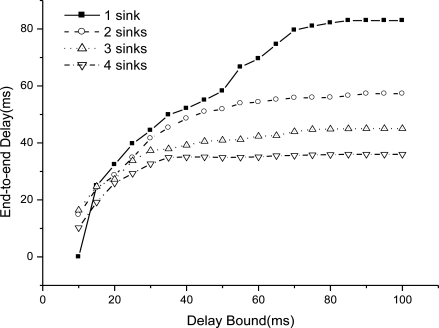
End-to-end delay as delay bound increases.

We simulate EAR performance when increase the sink number from 1 to 4 as the delay bound is set from 10 to 100. Figure 5 shows the end-to-end delay as delay bound increases.

**Figure 6.**
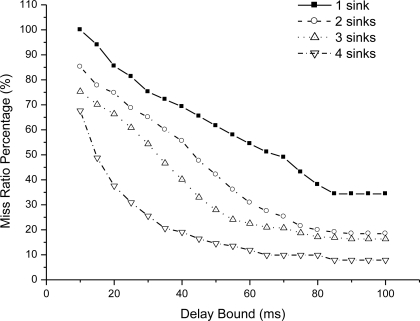
Miss ratio percentage as delay bound increases.

**Figure 7.**
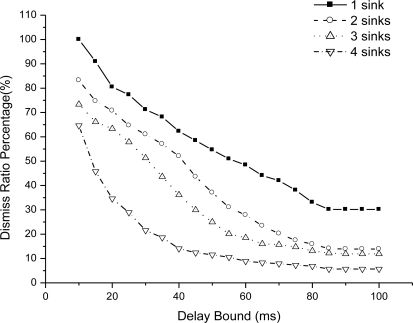
Dismiss ratio percentage as delay bound increases.

**Figure 8.**
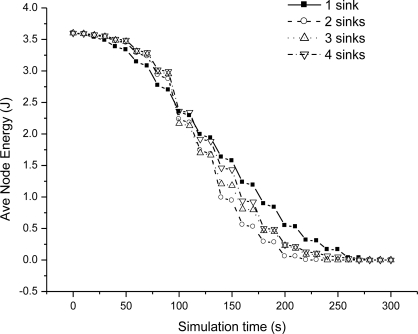
Average node energy when delay bound = 70 ms.

We could see that end-to-end delay decreases as the sink number increases, because more sinks incur more packet delivery within the bound. For a given number sink, the end-to-end delay increases slowly as we relax the bound. For one sink, the end-to-end delay is very small as the bound is 10 ms, because very seldom packet can be delivered within the bound. Figure 6 shows the miss ratio when we decrease delay bound. The packet miss ratio according to delay bound decreases as the sink number increases from 1 to 4. Because more sinks increases the real-time packet delivery probability. Figure 7 illustrates the packet dismiss ratio according to delay estimate. From the result, the dismiss ratio decreases as the sink number varies from 1 to 4. And we can see that EAR provides a good delay estimate and guide packet delivery towards the real-time direction when compared with miss ratio results in Figure 6. Figure 8 shows the average residual energy for node in the simulation time from 0 to 300 s when the delay bound is 70 ms. The average node energy does not vary greatly as we increase the number of sinks. Since increase the sink number, more packets are delivered by more energy consumption and also less routing trials with increased power. The node energy decreases as we relax the bound because more packets are delivered within the given delay bound.

### 6.2. EAR Performance Compared with No Power Adaptation and No Fire Situations

**Figure 9.**
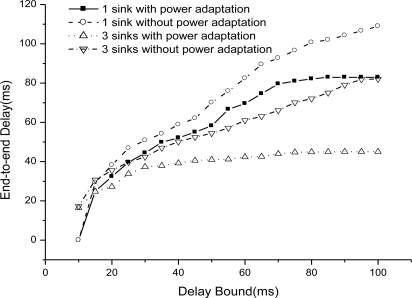
End-to-end delay with/without power adaptation.

We evaluate EAR routing when using 3 power levels adaptation and no power adaptation situations. Figure 9 illustrates the end-to-end delay with power adaptation and without power adaptation situations. We get the results with 1 sink and 3 sinks respectively. It is obviously the end-to-end delay decreases a lot when we use power adaptation. By the benefit of power level adaptation, we could increase the network connectivity in fire and also help to find lower delay route path to guarantee a real-time packet delivery under the given delay bound.

**Figure 10.**
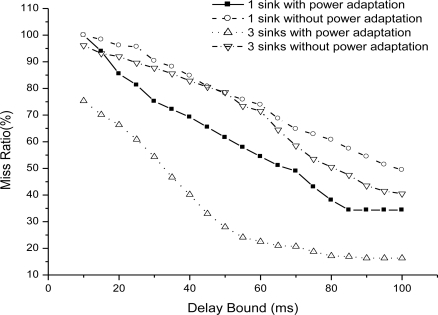
Miss ratio with/without power adaptation.

Figure 10 shows the miss ratio with/without power adaptation. The miss ratio increases greatly if we adapt power level in the network to increase the probability of real-time packet delivery.

**Figure 11.**
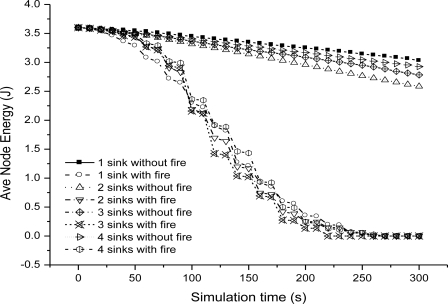
Average node energy when delay bound = 50 m.

We then evaluate energy efficiency of EAR routing when fire happens and no fire happens situations. Figure 11 illustrates the average node energy in the simulation time when delay bound is set to 50 ms. From the results, it is obvious that average node energy decreases in fire situation. But until 250 s of simulation, the average node energy is larger than 0. For delay bound chosen as 50 ms, the network is still effective until close to the end of the simulation in building fire situations.

### 6.3. Performance Compared with Other Protocols in Fire Hazard

**Figure 12.**
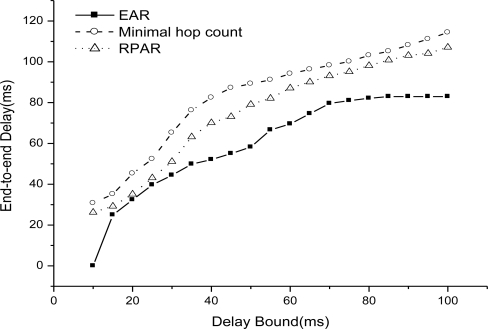
End-to-end delay as delay bound increases.

We then compare EAR with two related routing mechanisms: RPAR and minimal hop count routing. Figure 12 shows the end-to-end delay as delay bound increases from 10 to 100 ms when there is one sink (node 99). We can see that EAR has the minimal end-to-end delay as we relax the bound, then RPAR, and minimal hop count routing has the worst result.

Because EAR adapt power level to try to increase the probability of real-time delivery and it is adaptive to fire spreading by choosing the real-time route path avoiding the dangerous area in fire. RPAR also uses power adaptation to try to increase the real-time delivery, but they are not suitable for fired, and easily chooses a minimal delay path but in the fire area. There is no real-time guarantee mechanism in minimal hop count routing and it is not suitable for fire situations.

**Figure 13.**
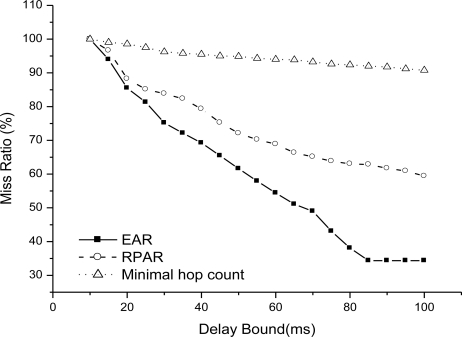
Miss ratio as delay bound increase.

**Figure 14.**
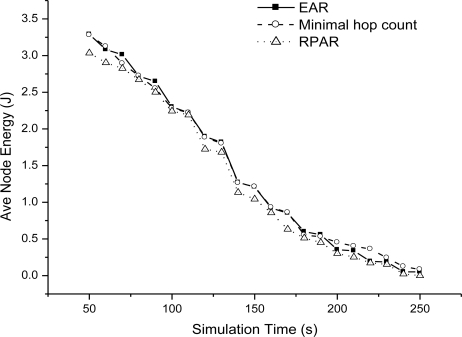
Average node energy when delay bound = 50 ms.

Figure 13 shows the miss ratio of real-time packet delivery with one sink. EAR achieves the best real-time data delivery. RPAR is not suitable for fire hazard. Even it adapts power level to try to find a real-time delivery path, but the performance is bad in fire. Figure 14 shows the average node energy in simulation time when delay bound is 50 ms. From the results, three routing mechanisms have similar energy efficiency. EAR has no obvious better energy efficiency, because it increase its power level to increase real-time packet delivery and incur energy consumption.

## 7. Conclusions and Future Work

We present a novel real-time and robust routing mechanism that is designed to be adaptive to emergency applications such as building fire hazards. The probability of end-to-end real-time communication is achieved by maintaining a desired delay based on a message propagation estimate and power level adaptation. The design is be adaptive to realistic hazard application characteristics including fires expanding, shrinking and diminishing. Our routing mechanism is designed as a localized protocol that makes decisions based solely on one-hop neighborhood information. Our ns-2 simulation results prove that the EAR routing mechanism achieves a good real-time packet delivery adaptive to fire emergency when compared with other related works. We have implemented our protocol into a 4-node TinyOS testbed. Future work will include implementation on a 100-node testbed we have deployed at our university to monitor and help to handle building fires.

## Figures and Tables

**Table 1.** Simulation parameters.

**Parameter**	**Value**
Propagation model	Shadowing
Shadowing deviation	4.0
Reference distance	1.0
PhyType	Phy/WirelessPhy/802_15_4
MacType	Mac/802_15_4
CSThresh_ (carrier sense threshold)	5.29754e-11
RXThresh_ (reception threshold)	5.29754e-11
Pt_(transmit power)	5.35395e-05/0.000214158/0.000481855
Freq_	2.4e+9
Traffic	CBR
Traffic packetSize_	70
Traffic Interval_	0.0969
Node Initial energy	3.6J
